# The Neonatal Development of Intraepithelial and Lamina Propria Lymphocytes in the Murine Small Intestine

**DOI:** 10.1155/1997/34891

**Published:** 1997

**Authors:** Jessica C. A. Ter Steege, Wim A. Buurman, Pierre-Philippe Forget

**Affiliations:** 1 Department of Pediatrics University Hospital Maastricht P.O. Box 5800 Maastricht 6202 AZ The Netherlands; 2 Department of Surgery University of Limburg FacII P.O. Box 616 Maastricht 6200 MD The Netherlands

**Keywords:** intestine, intraepithelial lymphocytes, lamina propria lymphocytes, mucosal immune system, ontogeny

## Abstract

During early neonatal life, important changes occur in the gut. The intestine is challenged by both milk and a microbial flora. Later on, at weaning, the diet of mice changes from milk to pelleted food leading to changes in microbial contents. This period seems essential for a complete development of the mucosal immune system. We investigated the development of both intraepithelial (IEL) and lamina propria lymphocytes (LPL), from day 5, and every 5 days, up to day 30 after birth. IEL and LPL were isolated from the small intestine and the phenotype was assessed by FACS analyses, using antibodies for detection of T-cell markers CD3, TCR*αβ*, TCR*γδ*, CD4, CD8*α*, CD8*β*, CD5, CD18, CD54, and CD49d. Our data show a clear increase in the number of LPL just before weaning, while the number of IEL increased after day 15. A more mature pattern of membrane antigen expression of both IEL and LPL was observed at weaning. The adhesion molecules CD18, CD54, and CD49d, essential for cellular communication of lymphocytes, showed an expression peak at weaning. In conclusion, the mouse mucosal immune system develops during the first 3 weeks of neonatal life leading to the formation of a more mature immune system at weaning.


*Developmental Immunology*, 1997, Vol. 5, pp. 121–128
Reprints available directly from the publisher
Photocopying permitted by license only

© 1997 OPA (Overseas Publishers Association)
Amsterdam B.V. Published under license in The Netherlands
under the Harwood Academic Publishers imprint
part of The Gordon and Breach Publishing Group
Printed in Malaysia

### The Neonatal Development of Intraepithelial and Lamina Propria Lymphocytes in the Murine Small Intestine

JESSICA C.A. TER STEEGE,*† WIM A. BUURMAN,‡ and PIERRE-PHILIPPE FORGET†

†*Department of Pediatrics, University Hospital Maastricht, P.O. Box 5800, 6202 AZ Maastricht, the Netherlands;* ‡*Department of Surgery, University of Limburg, FacII, P.O. Box 616, 6200 MD Maastricht, the Netherlands*

*(Received 22 April, 1996; Accepted 3 July, 1996)*

During early neonatal life, important changes occur in the gut. The intestine is challenged by both milk and a microbial flora. Later on, at weaning, the diet of mice changes from milk to pelleted food leading to changes in microbial contents. This period seems essential for a complete development of the mucosal immune system. We investigated the development of both intraepithelial (IEL) and lamina propria lymphocytes (LPL), from day 5, and every 5 days, up to day 30 after birth. IEL and LPL were isolated from the small intestine and the phenotype was assessed by FACS analyses, using antibodies for detection of T-cell markers CD3, TCR$\alpha\beta$, TCR$\gamma\delta$, CD4, CD8$\alpha$, CD8$\beta$, CD5, CD18, CD54, and CD49d. Our data show a clear increase in the number of LPL just before weaning, while the number of IEL increased after day 15. A more mature pattern of membrane antigen expression of both IEL and LPL was observed at weaning. The adhesion molecules CD18, CD54, and CD49d, essential for cellular communication of lymphocytes, showed an expression peak at weaning. In conclusion, the mouse mucosal immune system develops during the first 3 weeks of neonatal life leading to the formation of a more mature immune system at weaning.

*Keywords:* intestine; intraepithelial lymphocytes; lamina propria lymphocytes; mucosal immune system; ontogeny

### INTRODUCTION

The gastrointestinal tract is one of the major sites of immunological challenge. Although a protective immune response against invading pathogenic microorganisms is essential, the systemic immune response to foreign dietary antigens must be suppressed. Therefore, in the intestine, perhaps more than in any other organ, immune reactivity must be tightly regulated. The question as to how the mucosal immune system categorizes antigens and selects a particular response is central to this process but remains largely unanswered (Strobel, 1990; Trejdosiewicz, 1993; Stokes et al., 1994).

The mucosal immune system mainly consists of two compartments with their own lymphocyte population separated by a basal membrane, the epithelium containing the intraepithelial lymphocytes (IEL) and the lamina propria containing the lamina propria lymphocytes (LPL). LPL are phenotypically and functionally similar to peripheral lymphocytes (Guy-Grand et al., 1991). Peyer's patches lymphocytes are considered as peripheral lymphocytes. Characterization of IEL has revealed a phe-

*Corresponding author.

notypic and functional heterogeneity that is further observed only in the thymus (Poussier and Julius, 1994b). Several lines of evidence have led to the conclusion that T-cell lymphopoiesis occurs within the intestinal epithelium, a tissue that shares the same endodermic origin as the thymus (Ferguson and Parrott, 1972; Bandeira et al., 1991; Rocha et al., 1991, 1994; Mosley and Klein, 1992; Poussier and Julius, 1994b). These data are in accordance with data that showed that, in mice, mRNA for Recombination Activating Gene 1 (RAG1) was present in $CD3^-$ IEL, suggesting that rearrangement of the gene to produce a functional TCR occurs in the intestine (Guy-Grand et al., 1992). Recently, RAG1 and RAG2 gene expression was also detected in the human intestine (Lynch et al., 1995).

Intestinal maturation seems partly regulated by internal triggers (Ferguson and Parrott, 1972; Diamond, 1986; Bandeira et al., 1990; Mosley and Klein, 1992), but complete maturation of the intestinal immune system seems to occur only after the intestine is challenged postnatally with both microbial and nutritional antigens (Bandeira et al., 1990). Maturation at weaning is also characterized by biochemical and morphological changes in the small intestine adapting the intestine to changes in the diet from milk to pelleted food (Henning and Kretchmer, 1973).

Detailed data on the mucosal immune system in mice have only been reported at the age of 1, 3, and 28 days (de Geus and Rozing, 1992) and in adult mice (Ferguson and Parrott, 1972; Guy-Grand et al., 1991; Goodman and Lefrancois, 1989). In the present study, we aimed at describing the mouse mucosal immune system ontogenesis by analyzing in detail the gut lymphocytes populations from day 5 onwards, and every 5 days, up to day 30.

### RESULTS

### Development of IEL

The number of IEL and $CD3^+$ IEL in the small intestine at the ages studied are shown in Fig. 1. On day 5, low numbers ($2.6 \times 10^5$) of IEL were isolated per small intestine of which only 17.5% were $CD3^+$ (see also Fig. 2). From day 5, the number of IEL increased, paralleled by a significant ($p < 0.01$) increase in the percentage of $CD3^+$ cells on day 15. Levels of 90% $CD3^+$ cells were reached on day 25 and stabilized thereafter. Before weaning (day 20), the intensity of the CD3 expression was low, however, from day 25, a large $CD3^{high}$ and a small $CD3^{low}$ population was detected (Fig. 3).

Next we studied the TCR gene usage during development. The data on TCR expression revealed that both

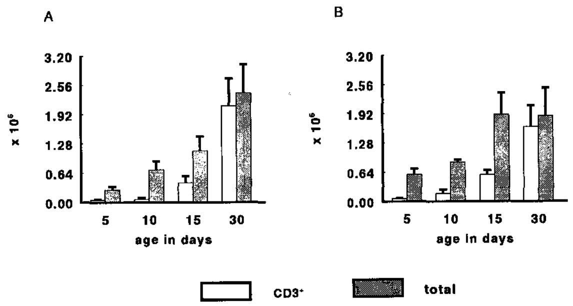

FIGURE 1 Total number and number of $CD3^+$ IEL (A) and LPL (B) recovered per small intestine increased during maturation. Data are given as mean ± SEM of six experiments.

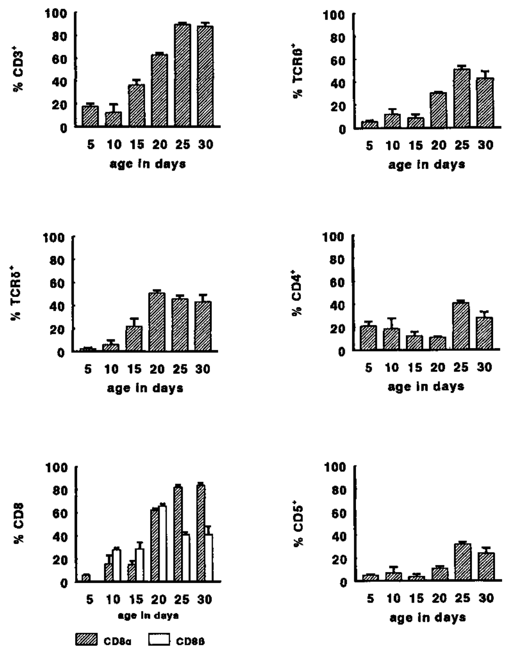

FIGURE 2 Phenotypic characterization of IEL during development. Data are given as percentage of total number of IEL recovered. At each time point, the mean ± SEM of six experiments is given.

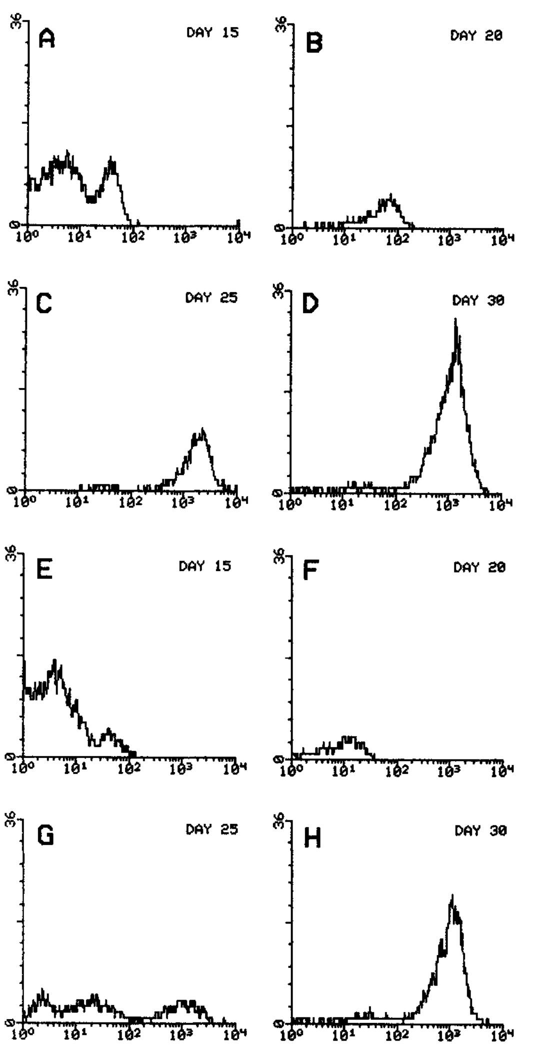

FIGURE 3 FACS analyses show an increase in intensity of CD3 expression on IEL (A, B, C, D) and LPL (E, F, G, H) during maturation of the mucosal immune system. The x axis gives the relative fluorescence intensity and the y axis the number of cells. Similar results were obtained in six experiments.

number and percentage of TCRγδ$^+$ thymic-independent IEL were very low shortly after birth (Fig. 2). The percentage TCRγδ$^+$ cells increased from day 10 onwards to 50% on day 20. From day 15, also the TCRαβ expression increased, reaching 50% on day 25.

In adult mice, the CD8 phenotype is characteristic of IEL. CD8 can consist of the thymus-independent αα homodimeric or the αβ heterodimeric thymus-dependent form. The number of CD8α$^+$ IEL increased rapidly from day 15 to reach adult levels on day 25. Double staining with CD8β revealed that on day 15, most of the CD8 cells consisted of a CD8αβ phenotype, and that on day 30, the thymic-independent CD8αα cells were more prominent (Fig. 2).

A minority of the IEL carried the thymus-dependent CD4 phenotype. The percentage of CD4$^+$ cells increased from 11% on day 20 to 30% on day 30 (Fig. 2). To further analyze the thymus-dependent population of IEL, we assessed CD5 membrane expression (Fig. 2). CD5 expression data revealed that the number of CD5$^+$ cells was very low (<7%) until day 20, and thereafter the percentage CD5$^+$ cells started to increase to reach 30% on day 25.

### Development of LPL

The population of LPL during the neonatal period was studied, simultaneously, in the same animals. On day 5, the number of LPL ($5.8 \times 10^5$) was higher than the number of IEL (Fig. 1), but the percentage of CD3 was similarly low (12%, Fig. 4). The percentage of CD3$^+$ LPL increased rapidly from day 5 to reach 90% on day 30. Similar to the intensity of CD3 expression in IEL, the intensity of the LPL CD3 expression increased with aging (Fig. 3).

Assessment of the TCR-type usage by LPL revealed an almost exclusive TCRαβ expression, similar to that of peripheral T cells (Fig. 4). No detectable TCRγδ expression was found in the lamina propria on the fifth postnatal day. A low percentage (10–15%) of TCRγδ was detected from day 10. In contrast to low TCRγδ expression, usage of TCRαβ increased rapidly to 60% on day 30.

In adult animals, the ratio of CD8 suppressor/cytotoxic to CD4-helper phenotype in the LPL population is similar to that of the peripheral T-cell population, being approximately 1:1 (Stokes et al., 1994). The percentage of CD8$^+$ LPL increased from day 5 rapidly to reach levels of 50% on day 25. Double staining for CD8α and CD8β revealed that the majority of the LPL expressed the thymus-dependent CD8αβ heterodimeric form (Fig. 4). The percentage of CD4$^+$ LPL reached levels of 40% on day 10 (Fig. 4). CD5 expression on

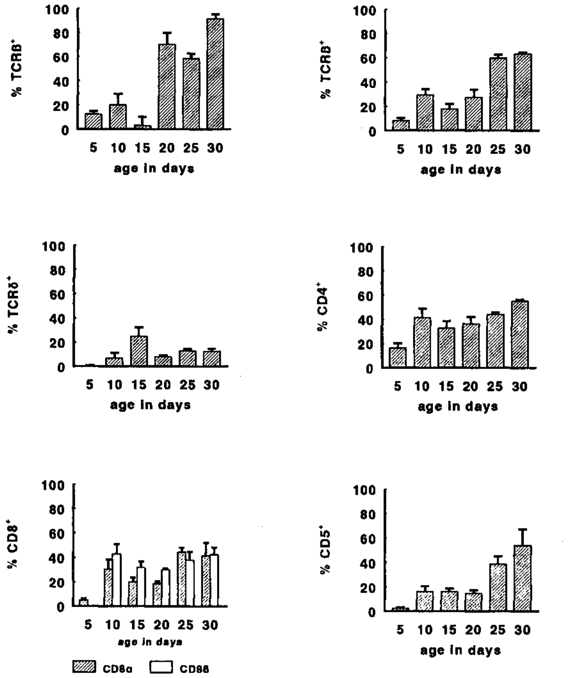

FIGURE 4 Phenotypic characterization of LPL during development. Data are given as percentage of total number of LPL recovered. At each time point, the mean ± SEM of six experiments is given.

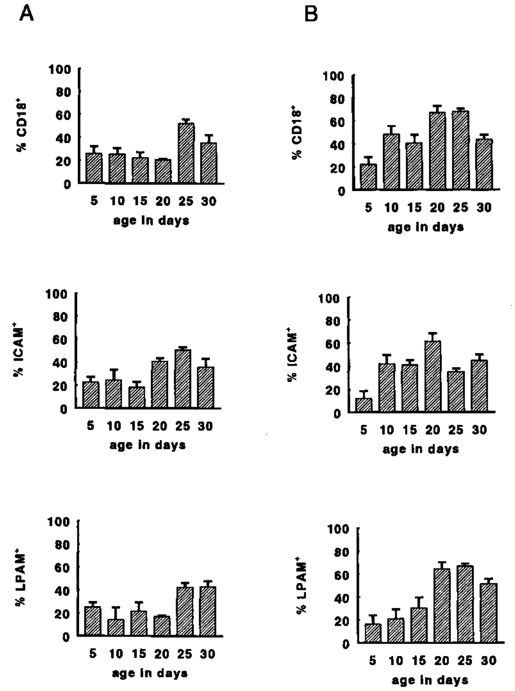

FIGURE 5 Adhesion molecule expression by (A) IEL and (B) LPL during development. Data are given as percentage of total IEL and LPL recovered. At each time point, the mean ± SEM of six experiments is given.

LPL showed an increase from day 20 to 54% $CD5^+$ LPL on day 30 (Fig. 4).

### Adhesion Molecule Expression of Mucosal Lymphocytes

Adhesion molecules play an important role in cellular communication and migration of lymphocytes and their microenvironment. In this perspective, we studied the kinetics of the expression of the adhesion molecules CD18, CD54, and CD49d during early neonatal life. The data on CD18 expression by IEL showed that 25% of the IEL express this adhesion molecule up to day 25, when the expression increased to 51% with a subsequent decrease to 35% on day 30 (Fig. 5). $CD54^+$ IEL were detected before weaning (20%) and increased to 40% after weaning. The homing receptor for the Peyer's patches, CD49d, was expressed on 20% of the IEL and increased to 40% on day 25.

The microenvironment and the origin of LPL differs substantially from IEL; it was therefore expected that the cellular expression of adhesion molecules would differ from that of IEL. CD18 expression on the LPL increased from day 5. A peak of 68% $CD18^+$ LPL was found at the age of 20 days, which subsequently decreased to 44% on day 30. Similarly, the percentage of $CD54^+$ LPL increased from day 5 to reach 61% on day 20 followed by a decrease to approximately 45% on day 30. The fraction of $CD49d^+$ LPL was 16% on day 5 and increased to 67% at the age of 25 days. A decrease to 52% was found at the age of 30 days (Fig. 5). From day 15, the level of expression for all the adhesion molecules was in general higher on the LPL population when compared to IEL.

### DISCUSSION

The data presented show that the murine intestine contains very low numbers of IEL and LPL shortly after birth. Similarly to the results in mice, the number of intestinal lymphocytes in young rats and pigs (5 days) were found to be 10% of that in adult animals (Lyscom and Brueton, 1983; Rothkötter et al., 1994). De Geus and Rozing (1992) reported a major increase in number of $CD3^+$ IEL and LPL between day 1 and 28. We show that the major increase in LPL takes place before weaning (between day 10 and 15) in contrast to the increase in IEL numbers, which takes place after day 15.

Another characteristic of mucosal lymphocytes in young mice is that both the number and the intensity of membrane antigen expression are very low. Consequently, during development, differentiation was characterized by both an increase in the number and intensity of membrane antigen expression.

In mature mice, both TCR$\alpha\beta$ and TCR$\gamma\delta$ expressing IEL are present. Although the extent of TCR$\alpha\beta$ and TCR$\gamma\delta$ expression is known to be strain-dependent (Poussier and Julius, 1994a), the presence of a large fraction of TCR$\gamma\delta^+$ cells, as detected in our experiments, is characteristic of murine IEL (Guy-Grand et al., 1991). On day 5, a very small percentage of $CD3^+$, TCR$\alpha\beta^+$, and TCR$\gamma\delta^+$ was detected, which increased in parallel with the increase in number of IEL. Interestingly, the increase in the percentage of TCR$\gamma\delta^+$ IEL was detected on day 15 before the increase in TCR$\alpha\beta^+$ IEL, which occurred at weaning (day 20). Our findings fits the hypothesis that TCR$\alpha\beta$ IEL, in contrast to TCR$\gamma\delta$, need antigen stimulation for their development in the intestinal epithelium because these cells seem to be stimulated by the change in diet occurring at weaning (Bandeira et al., 1990; De Geus et al., 1990; Machado et al., 1994; Rothkötter et al., 1994).

Next to the increase in the percentage of $CD8^+$ IEL, which started on day 20, weaning also affected CD8 expression. Whereas the thymus-dependent CD8$\alpha\beta$ form was more prominently expressed before weaning, the thymus-independent CD8$\alpha\alpha$ form increased rapidly at weaning, suggesting that luminal antigen stimulation favor the development of the thymus-independent CD8$\alpha\alpha$ form.

CD4 and CD5 expression increased somewhat later than CD8, in line with the observations of Lyscom and Brueton (1983) in rat. From day 25, the total percentage of $CD8^+$ and $CD4^+$ exceeded the percentage of $CD3^+$ IEL, indicating the presence of double-positive ($CD8^+$ $CD4^+$) IEL. Mosley et al. (1990) reported that approximately 50% of the $CD4^+$ IEL simultaneously expressed CD8. Both CD4 and CD5 are characteristic of thymus-dependent IEL. Their expression pattern was rather similar to that of TCR$\alpha\beta^+$ IEL.

The LPL population is known to be phenotypically and functionally different from IEL. LPL are thymus-dependent and are easily stimulated via the T-cell receptor (TCR) in contrast to IEL (Guy-Grand et al., 1991; Poussier et al., 1992; Poussier and Julius, 1994a). TCR expression of LPL revealed a low expression (10%) of TCR$\gamma\delta$ and a high expression of the $\alpha\beta$ TCR in 30-day-old mice. This TCR phenotype is characteristic of peripheral lymphocytes (Stokes et al., 1994a).

Adult CD4 expression was already reached on day 10. CD8$\alpha\beta$ and CD5 LPL were also present at this age but the increase to adult levels was found at weaning. In 30-day-old mice, the LPL CD8 suppressor/cytotoxic and CD4 helper expression is about 1:1, which is characteristic for peripheral T cells (Stokes et al., 1994).

Migration of lymphocytes to the intestine, mediated by adhesion molecules, plays an essential role in the development of the intestinal immune system (Duijvestijn and Hamann, 1989; Holzmann et al., 1989). From this perspective, we investigated the development of the expression of the adhesion molecules CD18, CD54, and CD49d during neonatal life.

The expression of the $\beta_2$ integrin CD18 was low on murine IEL, peaking on day 25. In contrast to IEL, we found a much higher expression on murine LPL, similar to what has been reported in humans (Jarry et al., 1990; Smart et al., 1991). CD54 expression on IEL was about 20% and increased around weaning in parallel with an increase in the number of IEL, probably indicating IEL activation. CD54 on LPL also showed an expression peak around weaning, albeit earlier than for IEL.

As expected, only a small proportion of the IEL expressed the $\alpha4\beta7$ integrin CD49d, homing receptor for the Peyer's patches (Holzmann et al., 1989, Boll et al., 1995), indicating that a small population of IEL migrated from the periphery. CD49d expression on LPL increased rapidly around weaning, suggesting an increase of LPL homing to the Peyer's patches. Boll and Reimann (1995) reported similar levels of CD49d

(LPAM) expression in adult mice and Trejdosiewicz (1992) in humans.

In conclusion, our data show that not only the phenotype of IEL and LPL are different, but also the kinetics of changes in phenotype during development are different. LPL are present in the intestine early after birth and are almost completely differentiated before weaning in contrast to IEL, which develop completely after weaning (day 20). The peak in expression of adhesion molecules around weaning indicate an important role during the development of the mucosal immune system especially in the lamina propria because adhesion molecule expression was higher for LPL than for IEL.

### MATERIAL AND METHODS

### Reagents and Antibodies

EDTA, dithiothreitol (DTT), collagenase (Type IV), and bovine serum albumin (BSA) were obtained from Sigma (St. Louis, MO). Medium consisted of RPMI1640 (Gibco BRL, Paisley, UK) supplemented with 10% heat-inactivated fetal calf serum (Hyclone, Logan, UT), 100 U/ml penicillin, 100 μg/ml streptomycin, 200 mM glutamine (Gibco BRL), and 0.1 mg/ml DNAse (Sigma). Percoll was obtained from Pharmacia (Uppsala, Sweden), biotin-X-NHS from Calbiochem (La Jolla, CA), and streptavidin phycoerythrin (RPE) from Caltag (SO San Francisco, CA).

Antibodies were obtained from hybridoma lines GK1.5 (rat anti-mouse CD4, IgG2b) (Dialynas et al. 1983), 53–6.72 (rat anti-mouse CD8α, IgG2a) (Ledbetter and Herzenberg, 1979), YN1/1.74 (rat anti-mouse CD54 (ICAM), IgG2a) (Takei, 1985), R1-2 (rat anti-mouse CD49d (α4β7, LPAM), IgG2b) (Holzmann et al., 1989), 2E6 (hamster anti-mouse CD18, IgG) (Metlay et al., 1990), H57-597 (hamster anti-mouse TcRβ, IgG) (Kubo et al., 1989) all from the ATCC (Rockville, MD). Hybridoma cell line 145-2C11 (hamster anti-mouse CD3, IgG) (Oberdan et al., 1987) was a gift of Dr. Bluestone (National Cancer Institute, Bethesda, MD). Hybridoma GL3 (hamster anti-mouse TcRγ, IgG) (Goodman and Lefrancois, 1989) was kindly provided by Dr. L. Lefrancois (UNCONN Health Center, Farmington, CT). Anti-mouse CD8β (Yb 156.7.7, IgG2b) (Qin et al., 1989) and CD5 (YTS 121.5.2, IgG2b) (Cobbold et al., 1984) antibodies were a gift of Dr. S. P. Cobbold (Department of Pathology, Oxford, UK). Monoclonal antibodies (mabs) were purified from culture supernatant by affinity chromatography. All antibodies were conjugated with biotin.

### Animals

Swiss mice were obtained from Charles River Breeding Laboratories (Heidelberg, FRG). They were maintained on a standard laboratory diet and were allowed free access to water. Guidelines of the Committee for Care and Use of Laboratory Animals from the University of Limburg were followed throughout. Litters were reduced to 10 pups per lactating mother, with free access to the nipples. The mice were weaned on day 20. From day 15, they started to eat food pellets.

### Isolation of Intestinal Lymphocytes

IEL and LPL were isolated using a modification of the method of Van der Heijden and Stok (1987). In short, the small bowel was immediately removed from the stomach to the appendix. The intestine was flushed with PBS (4°C) to remove fecal content and trimmed of fat, mesentery, and Peyer's patches. Subsequently, the intestine was cut longitudinally, washed twice with PBS supplemented with 50 mM glucose, and cut in pieces of 0.5–1.0 cm. IEL were isolated by incubation of the pieces of intestine in an EDTA/DTT solution for 15 min after which the supernatant was removed and the pieces were incubated in medium for 30 min at 37°C. Next, the cell suspensions obtained were passed over a 50 μm nylon gauze, centrifuged (5 min, 370 g), and resuspended in medium. The pieces of the intestine remaining on the nylon gauze were used for isolation of LPL by incubation in 20 ml medium supplemented with 7,000 units collagenase in a shaking waterbath at 37°C for 30 min. This procedure was repeated once after the supernatant with the freed cells was removed. Next, the cell suspensions obtained were passed over a 50-μm gauze, pelleted, and resuspended in medium. A further purification of the lymphocytes (both IEL and LPL) was obtained by density gradient centrifugation of the obtained epithelial and lamina propria cell fractions, using a discontinuous percoll gradient. The viability was always over 90% as assessed by trypan blue exclusion. Cells were kept at 4°C until further processing.

### FACS Analysis

For FACS analysis of young animals (<day 20), intestines of three mice were pooled to obtain sufficient numbers of lymphocytes for FACS staining. Cells ($10^5$ in 0.1 ml) were washed with PBS containing 0.1% BSA and azide and incubated with mab at the appropriate dilution (1–10 μg/ml) in PBS containing 0.1% BSA and azide for 1 hr at 4°C. Subsequently, the cells were washed three times with cold PBS containing 0.1% BSA and azide (4°C) and incubated with streptavidin-phycoerythrin for 1 hr (4°C). After this incubation, the cells were washed three times and analyzed using a FACSsort equiped with the LYSES II program (Becton and Dickinson, Sunnyvale, CA). Cells were gated out using forward versus side scatter to exclude aggregates, dead cells, or debris. Fluorescence intensity was expressed as mean channel number.

### *Acknowledgment*

This work was supported by a Grant from the Nutricia Research, Zoetermeer, the Netherlands.

### *References*

Bandeira, A., Itohara, S., Bonneville, M., Burlen-Defranoux, O., Mota-Santos, T., Coutinho, A. and Tonegawa, S. (1991) Extrathymic origin of intestinal intraepithelial lymphocytes bearing T-cell antigen receptor γδ. *Proc. Natl. Acad. Sci. USA*, **88**: 43–47.

Bandeira, A., Mota-Santos, T., Itohara, S., Degermann, S., Huesser, C., Tonegawa, S. and Coutinho, A. (1990) Localization of γ/δ T cells to the intestinal epithelium is independent of normal microbial colonization. *J. Exp. Med.*, **172**: 239–244.

Boll, G. and Reimann, J. (1995) Lamina propria T cell subsets in the small and large intestine of euthymic and athymic mice. *Scand. J. Immunol.*, **42**: 191–201.

Boll, G., Rudolphi, A., Spieβ, S. and Reimann, J. (1995) Regional specialization of intraepithelial T cells in the murine small and large intestine. *Scand. J. Immunol.*, **41**: 103–113.

Cobbold, S. P., Jayasuriya, A., Nash, A., Prospero, T. D. and Waldmann, H. (1984) Therapy with monoclonal antibodies by elimination of T-cell subsets in vivo. *Nature*, **312**: 548–551.

De Geus, B. and Rozing, J. (1992) Co-expression of TCR-β and TCR-δ chains on intestinal lamina propria lymphocytes during neonatal development in euthymic and athymic mice. In Differentiation and Characterization of Murine Intestinal Intraepithelial Lymphocytes, thesis (University of Leiden, Leiden), pp. 90–109.

De Geus, B., Shultz, L. D., van den Enden, M., Coolen, C. and Rozing, J. (1990) Analysis of intestinal intraepithelial lymphocytes in athymic (nude) and scid mice. In Advances in Mucosal Immunology, McDonald T. T., et al. Ed. (Dordrecht: Kluwer Academic Publishers), p. 71.

Dialynas, D. P., Wilde, D. B., Marrack, P., Pierres A., Wall, K. A., Harvan, W., Otten, G., Loken, M. R., Pierres, M., Kappler, J. and Fitch, F. W. (1983) Characterization of the murine antigenic determinant, designated L3T4a, recognized by monoclonal antibody GK1.5: Expression of L3T4a by functional T cell clones appears to correlate primarily with class II MHC antigen reactivity. *Immunol. Rev.*, **74**: 29–56.

Diamond, J. M. (1986) Hard-wired local triggering of intestinal enzyme expression. *Nature*, **324**: 408.

Duijvestijn, A. and Hamann, A. (1989) Mechanisms and regulation of lymphocyte migration. *Immunol. Today*, **10**: 23–28.

Ferguson, A. and Parrott, D. M. (1972) Growth and development of "antigen-free" grafts of foetal mouse intestine. *J. Pathol.*, **106**: 95–101.

Goodman, T. and Lefrancois, L. (1989) Intraepithelial lymphocytes. Anatomical site, not T cell receptor form, dictates phenotype and function. *J. Exp. Med.*, **170**: 1569–1581.

Guy-Grand, D., Cerf-Bensussan, N., Malissen, B., Malassis-Seris, M., Briottet, C. and Vassalli, P. (1991) Two gut intraepithelial $CD8^+$ lymphocyte populations with different T cell receptors: A role for the gut epithelium in T cell differentiation. *J. Exp. Med.*, **173**: 471–481.

Guy-Grand, D., Vanden Broecke, C., Briottet, C., Malassis-Seris, M., Selz, F. and Vassalli, P. (1992) Different expression of the recombination activity gene RAG-1 in various populations of thymocytes, peripheral T cells and gut thymus-independent intraepithelial lymphocytes suggests two pathways of T cell receptor rearrangement. *Eur. J. Immunol.*, **22**: 505–510.

Henning, S. J. and Kretchmer, N. (1973) Development of intestinal function in mammals. *Enzyme*, **15**: 3–23.

Holzmann, B., McIntyre, B. W. and Weissman, I. L. (1989) Identification of a murine Peyer's patch-specific lymphocyte homing receptor as an integrin molecule with an α chain homologous to human VLA-4α. *Cell*, **56**: 37–46.

Jarry, A., Cerf-Bensussan, N., Brousse, N., Selz, F. and Guy-Grand, D. (1990) Subsets of $CD3^+$ (T cell receptor α/β or γ/δ) and $CD3^-$ lymphocytes isolated from normal human gut epithelium display phenotypical features different from their counterparts in peripheral blood. *Eur. J. Immunol.*, **20**: 1097–1103.

Kubo, R. T., Born, W., Kappler, J. W., Marrack, P. and Pigeon, M. (1989) Characterization of a monoclonal antibody which detects all murine αβ T cell receptors. *J. Immunol.*, **142**: 2736–2742.

Ledbetter, J. A. and Herzenberg, L. A. (1979) Xenogeneic monoclonal antibodies to mouse lymphoid differentiation antigens. *Immunol. Rev.*, **47**: 63–90.

Lynch, S., Kelleher, D., McManus, R. and O'Farrelly, C. (1995) RAG1 and RAG2 expression in human intestinal epithelium: Evidence of extrathymic T cell differentiation. *Eur. J. Immunol.*, **25**: 1143–1147.

Lyscom, N. and Brueton, M. J. (1983) The development of intraepithelial and Peyer's patch lymphocyte sub-types in the small intestine of newborn rats. *Clin. Exp. Immunol.*, **54**: 158–162.

Machado, C. S. M., Rodrigues, M. A. M. and Maffei, H. V. L. (1994) Assessment of gut intraepithelial lymphocytes during late gestation and the neonatal period. *Biol. Neonate.*, **66**: 324–329.

Metlay, J. P., Witmer-Pack, M. D., Agger, R., Crowley, M. T., Lawless, D. and Steinman, R. M. (1990) The distinct leukocyte integrins of mouse spleen dendritic cells as identified with new hamster monoclonal antibodies. *J. Exp. Med.*, **171**: 1753–1771.

Mosley, R. L. and Klein, J. R. (1992) Peripheral engraftment of fetal intestine into athymic mice sponsors T cell development: Direct evidence for thymopoietic function of murine small intestine. *J. Exp. Med.*, **176**: 1365–1373.

Mosley, R. L., Styre, D. and Klein, J. R. (1990) $CD4^+$ $CD8^+$ murine intestinal intraepithelial lymphocytes. *Int. Immunol.*, **2**: 361–365.

Oberdan, L., Foo, M., Sachs, D. H., Samelson, L. E. and Bluestone, J. A. (1987) Identification of a monoclonal antibody specific for a murine T3 polypeptide. *Proc. Natl. Acad. Sci.* USA, **84**: 1374–1378.

Poussier, P., Edouard, P., Lee, C., Binnie, M. and Julius, M. (1992) Thymus-independent development and negative selection of T cells expressing T cell receptor $\alpha/\beta$ in the intestinal epithelium: Evidence for distinct circulation patterns of gut- and thymus-derived T lymphocytes. *J. Exp. Med.*, **176**: 187–199.

Poussier, P. and Julius, M. (1994a) Thymus independent T cell development and selection in the intestinal epithelium. *Annu. Rev. Immunol.*, **12**: 521–553.

Poussier, P. and Julius, M. (1994b) Intestinal intraepithelial lymphocytes: The plot thickens. *J. Exp. Med.*, **180**: 1185–1189.

Qin, S., Cobbold S., Benjamin, R. and Waldmann, H. (1989) Induction of classical transplantation tolerance in the adult. *J. Exp. Med.*, **169**: 779–794.

Rocha B., Vassalli, P. and Guy-Grand, D. (1991) The V$\beta$ repertoire of mouse gut homodimeric $\alpha$ $CD8^+$ intraepithelial T cell receptor $\alpha/\beta^+$ lymphocytes reveals a major extrathymic pathway of T cell differentiation. *J. Exp. Med.*, **173**: 483–486.

Rocha, B., Vassalli, P. and Guy-Grand, D. (1994) Thymic and extrathymic origins of gut intraepithelial lymphocyte populations in mice. *J. Exp. Med.*, **180**: 681–686.

Rothkötter, H. J., Kirchhoff, T. and Pabst, R. (1994) Lymphoid and nonlymphoid cells in the epithelium and lamina propria of intestinal mucosa of pigs. *Gut*, **35**: 1582–1589.

Smart, C. J., Calabrese, A., Oakes, D. J., Howdle, P. D. and Trejdosiewicz, L. K. (1991) Expression of the LFA-1 $\beta_2$ integrin (CD11a/CD18) and ICAM-1 (CD54) in normal and coeliac small bowel mucosa. *Scand. J. Immunol.*, **34**: 299–305.

Strobel, S. (1990) Immunologically mediated damage to the intestinal mucosa. *Acta Paediatr. Scand. Suppl.*, **365**: 46–57.

Stokes, C. R., Bailey, M. and Wilson, A. D. (1994) Immunology of the pocrine gastrointestinal tract. *Veter. Immunol. Immunopath.*, **43**: 143–150.

Takei, F. (1985) Inhibition of mixed lymphocyte response by a rat monoclonal antibody to a novel murine lymphocyte activation antigen (Mala-2). *J. Immunol.*, **134**: 1403–1407.

Trejdosiewicz, L. K. (1992) Intestinal intraepithelial lymphocytes and lymphoepithelial interactions in the human gastrointestinal mucosa. *Immunol. Lett.*, **32**: 13–20.

Trejdosiewicz, L. K. (1993) What is the role of human intestinal intraepithelial lymphocytes? *Clin. Exp. Immunol.*, **94**: 395–397.

Van der Heijden, P. J. and Stok, W. (1987) Improved procedure for the isolation of functionally active lymphoid cells from the murine intestine. *J. Immunol. Meth.*, **103**: 161–167.

